# 7-Isopropyl-1,4a-dimethyl-1,2,3,4,4a,5,6,7,8,9,10,10a-dodeca­hydro­phenan­threne-1-carboxylic acid

**DOI:** 10.1107/S1600536809042305

**Published:** 2009-10-23

**Authors:** Xiao-Ping Rao, Zhan-Qian Song, Shi-Bin Shang, Yong Wu

**Affiliations:** a Institute of Chemical Industry of Forest Products, Chinese Academy of Forestry, Nanjing 210042, People’s Republic of China

## Abstract

The title compound, C_20_H_32_O_2_, has been isolated from hydrogenated rosin. There are two independent mol­ecules in the asymmetric unit. In each mol­ecule, the cyclo­hexane ring assumes a chair conformation, while the two cyclo­hexene rings adopt half-chair and envelope conformations. Inter­molecular O—H⋯O hydrogen bonding between carboxyl groups links pairs of independent mol­ecules into dimers.

## Related literature

For the applications of pine resin acids, see: Piispanen *et al.* (2001 ▶); Jia *et al.* (2009 ▶); Sepulveda *et al.* (2005 ▶); Rao, Song & He (2008 ▶); Rao, Song, He & Jia (2008 ▶); Bicu & Mustata (2000 ▶); Hoa *et al.* (1993 ▶).
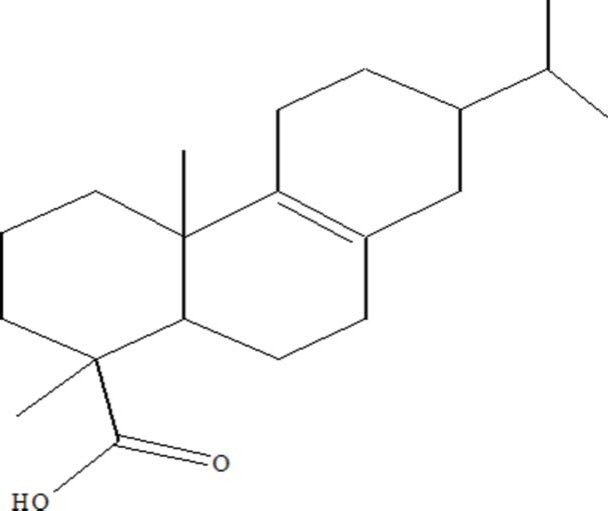


## Experimental

### 

#### Crystal data

C_20_H_32_O_2_
*M* *_r_* = 304.42
Monoclinic, 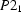

*a* = 11.543 (2) Å
*b* = 13.580 (3) Å
*c* = 13.345 (3) Å
β = 114.93 (3)°
*V* = 1897.0 (7) Å^3^
*Z* = 4
Mo *K*α radiation
μ = 0.07 mm^−1^
*T* = 293 K
0.30 × 0.20 × 0.10 mm

#### Data collection

Enraf–Nonius CAD-4 diffractometer
Absorption correction: none
3775 measured reflections
3590 independent reflections
2046 reflections with *I* > 2σ(*I*)
*R*_int_ = 0.025
3 standard reflections every 200 reflections intensity decay: 1%

#### Refinement

*R*[*F* ^2^ > 2σ(*F* ^2^)] = 0.062
*wR*(*F* ^2^) = 0.194
*S* = 1.00
3590 reflections
385 parameters
1 restraint
H-atom parameters constrained
Δρ_max_ = 0.41 e Å^−3^
Δρ_min_ = −0.18 e Å^−3^

### 

Data collection: *CAD-4 Software* (Enraf–Nonius, 1985 ▶); cell refinement: *CAD-4 Software*; data reduction: *XCAD4* (Harms & Wocadlo, 1995 ▶); program(s) used to solve structure: *SHELXTL* (Sheldrick, 2008 ▶); program(s) used to refine structure: *SHELXTL*; molecular graphics: *SHELXTL*; software used to prepare material for publication: *SHELXTL*.

## Supplementary Material

Crystal structure: contains datablocks I, global. DOI: 10.1107/S1600536809042305/xu2627sup1.cif

Structure factors: contains datablocks I. DOI: 10.1107/S1600536809042305/xu2627Isup2.hkl

Additional supplementary materials:  crystallographic information; 3D view; checkCIF report

## Figures and Tables

**Table 1:** Hydrogen-bond geometry (Å, °)

*D*—H⋯*A*	*D*—H	H⋯*A*	*D*⋯*A*	*D*—H⋯*A*
O2—H2*D*⋯O3^i^	0.82	1.82	2.632 (6)	170
O4—H4*B*⋯O1^ii^	0.82	1.82	2.638 (6)	173

Symmetry codes: (i) 
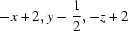
; (ii) 
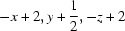
.

**Fig. 1.**
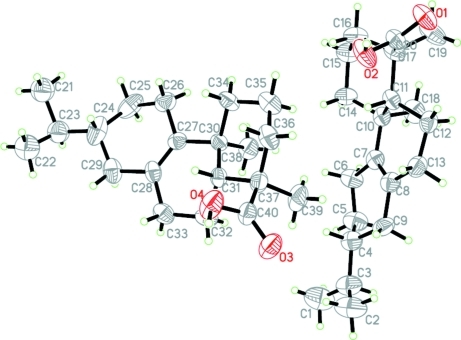
The molecular structure of the title compound with atom labels. Displacement ellipsoids were drawn at the 30% probability level.

## supplementary crystallographic information

### Comment

Pine resin acids are natural diterpenoid compounds, monocarboxylic acids of alkylated hydrophenanthrene acids constitutes the principal resin acid. Pine resin acids are widely used as starting material for design and synthesis of surfactants (Piispanen *et al.*, 2001; Jia *et al.*, 2009), biological compounds (Sepulveda *et al.*, 2005; Rao *et al.*, 2008*a*,b) and polymers (Bicu *et al.*, 2000; Hoa *et al.*, 1993). In this work we describe the crystal structure of the title compound.

Two crystallorgraphica independent molecules exist in the title structure, in each molecule there are three six-membered rings, in which they form half-chair, envelope and chair conformations, respectively (Fig. 1). The two methyl groups in the same side of tricyclo phenanthrene structure. The crystal structure is stabilized by intermolecular O—H···O hydrogen bonds (Table 1).

### Experimental

The title compound was isolated from hydrogenated rosin by recrystallization 5 times from acetone. Single crystals were grown from acetone.

### Refinement

H atoms were positioned geometrically and refined as riding atoms with C—H = 0.96 Å and *U*_iso_(H) = 1.5*U*_eq_(C) for methyl H atoms, and C—H = 0.97 - 0.98 Å and *U*_iso_(H) = 1.2*U*_eq_(C) for the other H atoms.

### Crystal data

**Table:**

C_20_H_32_O_2_	*F*(000) = 672
*M**_r_* = 304.42	*D*_x_ = 1.066 Mg m^−^^3^
Monoclinic, *P*2_1_	Mo *K*α radiation, λ = 0.71073 Å
Hall symbol: P 2yb	Cell parameters from 25 reflections
*a* = 11.543 (2) Å	θ = 10–13°
*b* = 13.580 (3) Å	µ = 0.07 mm^−^^1^
*c* = 13.345 (3) Å	*T* = 293 K
β = 114.93 (3)°	Block, colorless
*V* = 1897.0 (7) Å^3^	0.30 × 0.20 × 0.10 mm
*Z* = 4	

### Data collection

**Table:**

Enraf–Nonius CAD-4 diffractometer	*R*_int_ = 0.025
Radiation source: fine-focus sealed tube	θ_max_ = 25.3°, θ_min_ = 1.7°
graphite	*h* = 0→13
ω/2θ scans	*k* = 0→16
3775 measured reflections	*l* = −16→14
3590 independent reflections	3 standard reflections every 200 reflections
2046 reflections with *I* > 2σ(*I*)	intensity decay: 1%

### Refinement

**Table:**

Refinement on *F*^2^	Primary atom site location: structure-invariant direct methods
Least-squares matrix: full	Secondary atom site location: difference Fourier map
*R*[*F*^2^ > 2σ(*F*^2^)] = 0.062	Hydrogen site location: inferred from neighbouring sites
*wR*(*F*^2^) = 0.194	H-atom parameters constrained
*S* = 1.00	*w* = 1/[σ^2^(*F*_o_^2^) + (0.113*P*)^2^] where *P* = (*F*_o_^2^ + 2*F*_c_^2^)/3
3590 reflections	(Δ/σ)_max_ = 0.002
385 parameters	Δρ_max_ = 0.41 e Å^−^^3^
1 restraint	Δρ_min_ = −0.18 e Å^−^^3^

### Special details

**Table:**

Geometry. All e.s.d.'s (except the e.s.d. in the dihedral angle between two l.s. planes) are estimated using the full covariance matrix. The cell e.s.d.'s are taken into account individually in the estimation of e.s.d.'s in distances, angles and torsion angles; correlations between e.s.d.'s in cell parameters are only used when they are defined by crystal symmetry. An approximate (isotropic) treatment of cell e.s.d.'s is used for estimating e.s.d.'s involving l.s. planes.
Refinement. Refinement of *F*^2^ against ALL reflections. The weighted *R*-factor *wR* and goodness of fit *S* are based on *F*^2^, conventional *R*-factors *R* are based on *F*, with *F* set to zero for negative *F*^2^. The threshold expression of *F*^2^ > σ(*F*^2^) is used only for calculating *R*-factors(gt) *etc*. and is not relevant to the choice of reflections for refinement. *R*-factors based on *F*^2^ are statistically about twice as large as those based on *F*, and *R*- factors based on ALL data will be even larger.

### Fractional atomic coordinates and isotropic or equivalent isotropic displacement parameters (Å^2^)

**Table:**

	*x*	*y*	*z*	*U*_iso_*/*U*_eq_	
O1	0.5995 (3)	−0.3368 (3)	0.9250 (4)	0.0953 (14)	
O2	0.7106 (3)	−0.2421 (4)	0.8667 (4)	0.1058 (16)	
H2D	0.7689	−0.2728	0.9145	0.159*	
C1	0.5526 (10)	0.4293 (8)	0.7003 (9)	0.162 (4)	
H1A	0.5698	0.4958	0.7268	0.242*	
H1B	0.4692	0.4258	0.6402	0.242*	
H1C	0.6155	0.4087	0.6752	0.242*	
C2	0.6790 (9)	0.3821 (7)	0.8947 (8)	0.151 (3)	
H2A	0.6744	0.3412	0.9515	0.226*	
H2B	0.6852	0.4499	0.9168	0.226*	
H2C	0.7530	0.3645	0.8830	0.226*	
C3	0.5574 (8)	0.3672 (6)	0.7861 (8)	0.117 (2)	
H3A	0.4866	0.3885	0.8031	0.140*	
C4	0.5336 (5)	0.2562 (5)	0.7585 (6)	0.0872 (17)	
H4A	0.5989	0.2350	0.7342	0.105*	
C5	0.4077 (6)	0.2351 (5)	0.6649 (6)	0.101 (2)	
H5A	0.3967	0.2776	0.6030	0.122*	
H5B	0.3399	0.2497	0.6873	0.122*	
C6	0.3975 (5)	0.1284 (4)	0.6285 (5)	0.0835 (17)	
H6A	0.3094	0.1146	0.5788	0.100*	
H6B	0.4488	0.1192	0.5874	0.100*	
C7	0.4403 (4)	0.0550 (4)	0.7227 (5)	0.0673 (14)	
C8	0.5082 (5)	0.0867 (4)	0.8256 (5)	0.0743 (15)	
C9	0.5507 (6)	0.1922 (5)	0.8566 (6)	0.0921 (19)	
H9A	0.5022	0.2205	0.8938	0.111*	
H9B	0.6401	0.1926	0.9083	0.111*	
C10	0.4006 (4)	−0.0506 (4)	0.6951 (4)	0.0667 (14)	
C11	0.4961 (4)	−0.1151 (4)	0.7890 (4)	0.0656 (14)	
H11A	0.5800	−0.0982	0.7920	0.079*	
C12	0.5025 (5)	−0.0827 (5)	0.9023 (5)	0.0780 (16)	
H12A	0.4178	−0.0849	0.9009	0.094*	
H12B	0.5573	−0.1271	0.9596	0.094*	
C13	0.5550 (6)	0.0217 (5)	0.9272 (5)	0.0840 (17)	
H13A	0.5302	0.0508	0.9815	0.101*	
H13B	0.6476	0.0190	0.9590	0.101*	
C14	0.4079 (5)	−0.0832 (4)	0.5888 (4)	0.0774 (16)	
H14A	0.4900	−0.0634	0.5918	0.093*	
H14B	0.3423	−0.0487	0.5274	0.093*	
C15	0.3914 (6)	−0.1936 (5)	0.5660 (5)	0.0931 (19)	
H15A	0.3066	−0.2135	0.5565	0.112*	
H15B	0.3994	−0.2082	0.4981	0.112*	
C16	0.4894 (6)	−0.2508 (5)	0.6590 (5)	0.0857 (17)	
H16A	0.5736	−0.2339	0.6648	0.103*	
H16B	0.4761	−0.3206	0.6424	0.103*	
C17	0.4845 (5)	−0.2311 (4)	0.7703 (5)	0.0758 (16)	
C18	0.2594 (4)	−0.0591 (6)	0.6788 (5)	0.097 (2)	
H18A	0.2306	−0.1257	0.6600	0.145*	
H18B	0.2069	−0.0159	0.6203	0.145*	
H18C	0.2535	−0.0409	0.7460	0.145*	
C19	0.3683 (5)	−0.2784 (5)	0.7804 (6)	0.113 (2)	
H19A	0.3678	−0.3480	0.7674	0.169*	
H19B	0.2911	−0.2496	0.7268	0.169*	
H19C	0.3737	−0.2671	0.8533	0.169*	
C20	0.6034 (5)	−0.2739 (4)	0.8604 (5)	0.0715 (14)	
O3	1.0841 (3)	0.1724 (4)	0.9849 (3)	0.0991 (14)	
O4	1.1955 (3)	0.0884 (4)	0.9141 (3)	0.1037 (15)	
H4B	1.2551	0.1139	0.9661	0.156*	
C21	1.0284 (11)	−0.0278 (10)	0.1895 (9)	0.164 (2)	
H21A	1.0483	−0.0113	0.1286	0.246*	
H21B	0.9479	−0.0616	0.1625	0.246*	
H21C	1.0941	−0.0696	0.2400	0.246*	
C22	1.1466 (10)	0.0976 (10)	0.2801 (8)	0.164 (2)	
H22A	1.1628	0.1577	0.3218	0.246*	
H22B	1.1602	0.1082	0.2147	0.246*	
H22C	1.2035	0.0472	0.3243	0.246*	
C23	1.0201 (11)	0.0682 (10)	0.2501 (9)	0.164 (2)	
H23A	0.9582	0.1153	0.2004	0.197*	
C24	0.9939 (7)	0.0429 (7)	0.3525 (5)	0.110 (3)	
H24A	1.0632	0.0000	0.4007	0.132*	
C25	0.8671 (7)	−0.0108 (6)	0.3270 (5)	0.110 (3)	
H25A	0.7960	0.0328	0.2866	0.132*	
H25B	0.8601	−0.0680	0.2813	0.132*	
C26	0.8618 (6)	−0.0429 (5)	0.4348 (5)	0.0893 (18)	
H26A	0.7753	−0.0631	0.4191	0.107*	
H26B	0.9171	−0.0996	0.4639	0.107*	
C27	0.9020 (5)	0.0367 (4)	0.5217 (4)	0.0645 (13)	
C28	0.9620 (5)	0.1162 (4)	0.5122 (4)	0.0692 (14)	
C29	0.9967 (6)	0.1337 (6)	0.4162 (5)	0.0893 (19)	
H29A	0.9377	0.1813	0.3666	0.107*	
H29B	1.0817	0.1619	0.4442	0.107*	
C30	0.8702 (4)	0.0187 (4)	0.6208 (4)	0.0581 (12)	
C31	0.9664 (4)	0.0813 (4)	0.7182 (4)	0.0609 (13)	
H31A	1.0507	0.0599	0.7255	0.073*	
C32	0.9591 (5)	0.1894 (4)	0.6862 (5)	0.0763 (16)	
H32A	0.8724	0.2133	0.6626	0.092*	
H32B	1.0145	0.2281	0.7494	0.092*	
C33	1.0000 (5)	0.2002 (4)	0.5941 (5)	0.0804 (16)	
H33A	1.0923	0.2068	0.6259	0.097*	
H33B	0.9639	0.2606	0.5545	0.097*	
C34	0.8919 (6)	−0.0900 (4)	0.6569 (5)	0.0815 (16)	
H34A	0.8284	−0.1304	0.6004	0.098*	
H34B	0.9755	−0.1105	0.6637	0.098*	
C35	0.8836 (6)	−0.1070 (5)	0.7676 (5)	0.096 (2)	
H35A	0.7988	−0.0894	0.7599	0.115*	
H35B	0.8969	−0.1763	0.7867	0.115*	
C36	0.9802 (6)	−0.0478 (5)	0.8582 (5)	0.0916 (19)	
H36A	1.0648	−0.0696	0.8696	0.110*	
H36B	0.9707	−0.0602	0.9259	0.110*	
C37	0.9695 (5)	0.0636 (5)	0.8356 (4)	0.0731 (16)	
C38	0.7292 (4)	0.0467 (5)	0.5848 (5)	0.0867 (17)	
H38A	0.6762	0.0052	0.5248	0.130*	
H38B	0.7166	0.1143	0.5613	0.130*	
H38C	0.7068	0.0383	0.6459	0.130*	
C39	0.8541 (5)	0.1048 (6)	0.8505 (5)	0.101 (2)	
H39A	0.8626	0.0900	0.9236	0.152*	
H39B	0.7773	0.0753	0.7971	0.152*	
H39C	0.8502	0.1748	0.8400	0.152*	
C40	1.0881 (4)	0.1130 (5)	0.9177 (4)	0.0701 (14)	

### Atomic displacement parameters (Å^2^)

**Table:**

	*U*^11^	*U*^22^	*U*^33^	*U*^12^	*U*^13^	*U*^23^
O1	0.063 (2)	0.101 (3)	0.111 (3)	−0.009 (2)	0.026 (2)	0.039 (3)
O2	0.054 (2)	0.123 (4)	0.131 (3)	0.006 (2)	0.031 (2)	0.061 (3)
C1	0.158 (8)	0.104 (7)	0.171 (9)	−0.017 (6)	0.019 (7)	0.025 (7)
C2	0.145 (7)	0.095 (6)	0.184 (9)	−0.013 (6)	0.041 (7)	−0.028 (6)
C3	0.110 (5)	0.073 (5)	0.153 (7)	0.002 (4)	0.041 (5)	−0.014 (5)
C4	0.071 (3)	0.083 (5)	0.104 (4)	0.005 (3)	0.033 (3)	−0.004 (4)
C5	0.085 (4)	0.077 (5)	0.120 (5)	0.017 (3)	0.022 (4)	0.013 (4)
C6	0.064 (3)	0.073 (4)	0.089 (4)	0.005 (3)	0.008 (3)	0.009 (3)
C7	0.047 (2)	0.072 (4)	0.081 (4)	0.013 (2)	0.025 (3)	0.008 (3)
C8	0.069 (3)	0.071 (4)	0.079 (4)	0.015 (3)	0.027 (3)	0.003 (3)
C9	0.090 (4)	0.083 (4)	0.111 (5)	0.001 (3)	0.050 (4)	−0.029 (4)
C10	0.045 (2)	0.077 (4)	0.075 (3)	0.005 (3)	0.022 (2)	0.001 (3)
C11	0.047 (2)	0.078 (4)	0.068 (3)	0.000 (3)	0.022 (2)	0.014 (3)
C12	0.071 (3)	0.091 (4)	0.080 (4)	0.011 (3)	0.039 (3)	0.011 (3)
C13	0.092 (4)	0.091 (5)	0.075 (4)	0.007 (3)	0.042 (3)	−0.007 (3)
C14	0.072 (3)	0.069 (4)	0.069 (3)	−0.002 (3)	0.008 (3)	−0.005 (3)
C15	0.085 (4)	0.083 (5)	0.082 (4)	−0.008 (3)	0.007 (3)	−0.005 (4)
C16	0.100 (4)	0.062 (4)	0.074 (4)	−0.002 (3)	0.016 (3)	−0.003 (3)
C17	0.051 (3)	0.070 (4)	0.098 (4)	−0.012 (3)	0.023 (3)	0.006 (3)
C18	0.042 (3)	0.117 (5)	0.120 (5)	0.009 (3)	0.023 (3)	0.020 (4)
C19	0.063 (3)	0.100 (5)	0.146 (6)	−0.028 (3)	0.015 (4)	0.027 (5)
C20	0.060 (3)	0.072 (4)	0.083 (4)	−0.005 (3)	0.030 (3)	0.015 (3)
O3	0.072 (2)	0.134 (4)	0.094 (3)	−0.010 (2)	0.038 (2)	−0.040 (3)
O4	0.054 (2)	0.156 (4)	0.092 (3)	0.003 (2)	0.0222 (18)	−0.032 (3)
C21	0.170 (5)	0.192 (6)	0.159 (5)	0.045 (5)	0.098 (5)	0.032 (5)
C22	0.170 (5)	0.192 (6)	0.159 (5)	0.045 (5)	0.098 (5)	0.032 (5)
C23	0.170 (5)	0.192 (6)	0.159 (5)	0.045 (5)	0.098 (5)	0.032 (5)
C24	0.107 (5)	0.158 (8)	0.075 (4)	0.057 (5)	0.048 (4)	0.035 (5)
C25	0.118 (6)	0.131 (6)	0.064 (4)	0.035 (5)	0.022 (4)	−0.020 (4)
C26	0.087 (4)	0.092 (4)	0.087 (4)	−0.009 (3)	0.034 (3)	−0.025 (4)
C27	0.055 (3)	0.065 (3)	0.067 (3)	0.008 (3)	0.020 (2)	−0.004 (3)
C28	0.060 (3)	0.082 (4)	0.070 (3)	0.004 (3)	0.032 (2)	0.021 (3)
C29	0.083 (4)	0.114 (5)	0.075 (4)	0.013 (4)	0.037 (3)	0.032 (4)
C30	0.052 (2)	0.051 (3)	0.070 (3)	−0.007 (2)	0.025 (2)	0.000 (2)
C31	0.042 (2)	0.076 (4)	0.067 (3)	0.000 (2)	0.025 (2)	0.003 (3)
C32	0.064 (3)	0.055 (3)	0.093 (4)	−0.005 (3)	0.017 (3)	−0.004 (3)
C33	0.065 (3)	0.074 (4)	0.090 (4)	−0.008 (3)	0.020 (3)	0.006 (3)
C34	0.081 (4)	0.066 (4)	0.096 (4)	−0.026 (3)	0.035 (3)	−0.001 (3)
C35	0.099 (4)	0.079 (5)	0.111 (5)	−0.018 (4)	0.045 (4)	0.014 (4)
C36	0.093 (4)	0.097 (5)	0.089 (4)	−0.008 (4)	0.043 (4)	0.021 (4)
C37	0.061 (3)	0.090 (5)	0.073 (3)	−0.004 (3)	0.034 (3)	−0.005 (3)
C38	0.054 (3)	0.107 (5)	0.091 (4)	−0.013 (3)	0.022 (3)	−0.022 (4)
C39	0.054 (3)	0.154 (7)	0.102 (4)	0.001 (4)	0.039 (3)	−0.022 (4)
C40	0.059 (3)	0.092 (4)	0.061 (3)	−0.004 (3)	0.027 (2)	−0.008 (3)

### Geometric parameters (Å, °)

**Table:**

O1—C20	1.228 (6)	O3—C40	1.221 (6)
O2—C20	1.280 (6)	O4—C40	1.304 (6)
O2—H2D	0.8200	O4—H4B	0.8200
C1—C3	1.404 (12)	C21—C23	1.559 (16)
C1—H1A	0.9600	C21—H21A	0.9600
C1—H1B	0.9600	C21—H21B	0.9600
C1—H1C	0.9600	C21—H21C	0.9600
C2—C3	1.548 (11)	C22—C23	1.400 (14)
C2—H2A	0.9600	C22—H22A	0.9600
C2—H2B	0.9600	C22—H22B	0.9600
C2—H2C	0.9600	C22—H22C	0.9600
C3—C4	1.549 (10)	C23—C24	1.557 (11)
C3—H3A	0.9800	C23—H23A	0.9800
C4—C5	1.491 (9)	C24—C29	1.490 (11)
C4—C9	1.513 (9)	C24—C25	1.539 (11)
C4—H4A	0.9800	C24—H24A	0.9800
C5—C6	1.517 (9)	C25—C26	1.529 (9)
C5—H5A	0.9700	C25—H25A	0.9700
C5—H5B	0.9700	C25—H25B	0.9700
C6—C7	1.515 (8)	C26—C27	1.508 (8)
C6—H6A	0.9700	C26—H26A	0.9700
C6—H6B	0.9700	C26—H26B	0.9700
C7—C8	1.333 (7)	C27—C28	1.317 (7)
C7—C10	1.504 (8)	C27—C30	1.534 (7)
C8—C13	1.514 (8)	C28—C33	1.511 (8)
C8—C9	1.515 (9)	C28—C29	1.513 (7)
C9—H9A	0.9700	C29—H29A	0.9700
C9—H9B	0.9700	C29—H29B	0.9700
C10—C14	1.521 (8)	C30—C38	1.539 (7)
C10—C11	1.545 (7)	C30—C34	1.539 (8)
C10—C18	1.556 (7)	C30—C31	1.558 (7)
C11—C12	1.547 (8)	C31—C32	1.521 (8)
C11—C17	1.591 (8)	C31—C37	1.571 (7)
C11—H11A	0.9800	C31—H31A	0.9800
C12—C13	1.522 (9)	C32—C33	1.499 (8)
C12—H12A	0.9700	C32—H32A	0.9700
C12—H12B	0.9700	C32—H32B	0.9700
C13—H13A	0.9700	C33—H33A	0.9700
C13—H13B	0.9700	C33—H33B	0.9700
C14—C15	1.527 (9)	C34—C35	1.539 (9)
C14—H14A	0.9700	C34—H34A	0.9700
C14—H14B	0.9700	C34—H34B	0.9700
C15—C16	1.496 (8)	C35—C36	1.488 (9)
C15—H15A	0.9700	C35—H35A	0.9700
C15—H15B	0.9700	C35—H35B	0.9700
C16—C17	1.534 (8)	C36—C37	1.538 (9)
C16—H16A	0.9700	C36—H36A	0.9700
C16—H16B	0.9700	C36—H36B	0.9700
C17—C20	1.508 (8)	C37—C40	1.503 (7)
C17—C19	1.544 (8)	C37—C39	1.532 (7)
C18—H18A	0.9600	C38—H38A	0.9600
C18—H18B	0.9600	C38—H38B	0.9600
C18—H18C	0.9600	C38—H38C	0.9600
C19—H19A	0.9600	C39—H39A	0.9600
C19—H19B	0.9600	C39—H39B	0.9600
C19—H19C	0.9600	C39—H39C	0.9600
			
C20—O2—H2D	109.5	C40—O4—H4B	109.5
C3—C1—H1A	109.5	C23—C21—H21A	109.5
C3—C1—H1B	109.5	C23—C21—H21B	109.5
H1A—C1—H1B	109.5	H21A—C21—H21B	109.5
C3—C1—H1C	109.5	C23—C21—H21C	109.5
H1A—C1—H1C	109.5	H21A—C21—H21C	109.5
H1B—C1—H1C	109.5	H21B—C21—H21C	109.5
C3—C2—H2A	109.5	C23—C22—H22A	109.5
C3—C2—H2B	109.5	C23—C22—H22B	109.5
H2A—C2—H2B	109.5	H22A—C22—H22B	109.5
C3—C2—H2C	109.5	C23—C22—H22C	109.5
H2A—C2—H2C	109.5	H22A—C22—H22C	109.5
H2B—C2—H2C	109.5	H22B—C22—H22C	109.5
C1—C3—C2	113.2 (8)	C22—C23—C24	112.2 (9)
C1—C3—C4	116.6 (8)	C22—C23—C21	95.9 (9)
C2—C3—C4	110.5 (7)	C24—C23—C21	110.4 (9)
C1—C3—H3A	105.1	C22—C23—H23A	112.5
C2—C3—H3A	105.1	C24—C23—H23A	112.5
C4—C3—H3A	105.1	C21—C23—H23A	112.5
C5—C4—C9	109.9 (6)	C29—C24—C25	107.9 (5)
C5—C4—C3	113.5 (6)	C29—C24—C23	110.7 (8)
C9—C4—C3	114.0 (6)	C25—C24—C23	115.4 (7)
C5—C4—H4A	106.3	C29—C24—H24A	107.5
C9—C4—H4A	106.3	C25—C24—H24A	107.5
C3—C4—H4A	106.3	C23—C24—H24A	107.5
C4—C5—C6	111.6 (5)	C26—C25—C24	109.8 (5)
C4—C5—H5A	109.3	C26—C25—H25A	109.7
C6—C5—H5A	109.3	C24—C25—H25A	109.7
C4—C5—H5B	109.3	C26—C25—H25B	109.7
C6—C5—H5B	109.3	C24—C25—H25B	109.7
H5A—C5—H5B	108.0	H25A—C25—H25B	108.2
C7—C6—C5	114.1 (5)	C27—C26—C25	113.5 (6)
C7—C6—H6A	108.7	C27—C26—H26A	108.9
C5—C6—H6A	108.7	C25—C26—H26A	108.9
C7—C6—H6B	108.7	C27—C26—H26B	108.9
C5—C6—H6B	108.7	C25—C26—H26B	108.9
H6A—C6—H6B	107.6	H26A—C26—H26B	107.7
C8—C7—C10	123.1 (5)	C28—C27—C26	121.1 (5)
C8—C7—C6	119.2 (5)	C28—C27—C30	122.7 (5)
C10—C7—C6	117.7 (5)	C26—C27—C30	116.2 (5)
C7—C8—C13	124.5 (6)	C27—C28—C33	123.5 (5)
C7—C8—C9	124.7 (6)	C27—C28—C29	123.2 (6)
C13—C8—C9	110.8 (5)	C33—C28—C29	113.2 (5)
C4—C9—C8	113.4 (5)	C24—C29—C28	113.8 (6)
C4—C9—H9A	108.9	C24—C29—H29A	108.8
C8—C9—H9A	108.9	C28—C29—H29A	108.8
C4—C9—H9B	108.9	C24—C29—H29B	108.8
C8—C9—H9B	108.9	C28—C29—H29B	108.8
H9A—C9—H9B	107.7	H29A—C29—H29B	107.7
C7—C10—C14	112.0 (5)	C27—C30—C38	107.3 (4)
C7—C10—C11	107.6 (4)	C27—C30—C34	110.8 (4)
C14—C10—C11	106.9 (4)	C38—C30—C34	110.3 (4)
C7—C10—C18	107.3 (5)	C27—C30—C31	106.6 (4)
C14—C10—C18	108.3 (5)	C38—C30—C31	114.4 (4)
C11—C10—C18	114.7 (5)	C34—C30—C31	107.3 (4)
C10—C11—C12	110.8 (4)	C32—C31—C30	111.0 (4)
C10—C11—C17	116.8 (4)	C32—C31—C37	113.7 (4)
C12—C11—C17	113.7 (5)	C30—C31—C37	117.2 (4)
C10—C11—H11A	104.8	C32—C31—H31A	104.5
C12—C11—H11A	104.8	C30—C31—H31A	104.5
C17—C11—H11A	104.8	C37—C31—H31A	104.5
C13—C12—C11	109.2 (5)	C33—C32—C31	108.9 (5)
C13—C12—H12A	109.8	C33—C32—H32A	109.9
C11—C12—H12A	109.8	C31—C32—H32A	109.9
C13—C12—H12B	109.8	C33—C32—H32B	109.9
C11—C12—H12B	109.8	C31—C32—H32B	109.9
H12A—C12—H12B	108.3	H32A—C32—H32B	108.3
C8—C13—C12	112.9 (5)	C32—C33—C28	115.1 (4)
C8—C13—H13A	109.0	C32—C33—H33A	108.5
C12—C13—H13A	109.0	C28—C33—H33A	108.5
C8—C13—H13B	109.0	C32—C33—H33B	108.5
C12—C13—H13B	109.0	C28—C33—H33B	108.5
H13A—C13—H13B	107.8	H33A—C33—H33B	107.5
C10—C14—C15	114.8 (5)	C35—C34—C30	112.1 (5)
C10—C14—H14A	108.6	C35—C34—H34A	109.2
C15—C14—H14A	108.6	C30—C34—H34A	109.2
C10—C14—H14B	108.6	C35—C34—H34B	109.2
C15—C14—H14B	108.6	C30—C34—H34B	109.2
H14A—C14—H14B	107.5	H34A—C34—H34B	107.9
C16—C15—C14	111.0 (5)	C36—C35—C34	111.7 (5)
C16—C15—H15A	109.4	C36—C35—H35A	109.3
C14—C15—H15A	109.4	C34—C35—H35A	109.3
C16—C15—H15B	109.4	C36—C35—H35B	109.3
C14—C15—H15B	109.4	C34—C35—H35B	109.3
H15A—C15—H15B	108.0	H35A—C35—H35B	107.9
C15—C16—C17	112.9 (5)	C35—C36—C37	113.7 (5)
C15—C16—H16A	109.0	C35—C36—H36A	108.8
C17—C16—H16A	109.0	C37—C36—H36A	108.8
C15—C16—H16B	109.0	C35—C36—H36B	108.8
C17—C16—H16B	109.0	C37—C36—H36B	108.8
H16A—C16—H16B	107.8	H36A—C36—H36B	107.7
C20—C17—C16	108.2 (5)	C40—C37—C39	108.5 (5)
C20—C17—C19	107.6 (5)	C40—C37—C36	108.9 (5)
C16—C17—C19	113.5 (5)	C39—C37—C36	109.6 (5)
C20—C17—C11	105.6 (4)	C40—C37—C31	106.9 (4)
C16—C17—C11	106.8 (5)	C39—C37—C31	114.7 (5)
C19—C17—C11	114.7 (5)	C36—C37—C31	108.2 (5)
C10—C18—H18A	109.5	C30—C38—H38A	109.5
C10—C18—H18B	109.5	C30—C38—H38B	109.5
H18A—C18—H18B	109.5	H38A—C38—H38B	109.5
C10—C18—H18C	109.5	C30—C38—H38C	109.5
H18A—C18—H18C	109.5	H38A—C38—H38C	109.5
H18B—C18—H18C	109.5	H38B—C38—H38C	109.5
C17—C19—H19A	109.5	C37—C39—H39A	109.5
C17—C19—H19B	109.5	C37—C39—H39B	109.5
H19A—C19—H19B	109.5	H39A—C39—H39B	109.5
C17—C19—H19C	109.5	C37—C39—H39C	109.5
H19A—C19—H19C	109.5	H39A—C39—H39C	109.5
H19B—C19—H19C	109.5	H39B—C39—H39C	109.5
O1—C20—O2	120.6 (5)	O3—C40—O4	121.8 (5)
O1—C20—C17	122.6 (5)	O3—C40—C37	121.7 (5)
O2—C20—C17	116.8 (5)	O4—C40—C37	116.5 (5)

### Hydrogen-bond geometry (Å, °)

**Table:**

*D*—H···*A*	*D*—H	H···*A*	*D*···*A*	*D*—H···*A*
O2—H2D···O3^i^	0.82	1.82	2.632 (6)	170
O4—H4B···O1^ii^	0.82	1.82	2.638 (6)	173

Symmetry codes: (i) −*x*+2, *y*−1/2, −*z*+2; (ii) −*x*+2, *y*+1/2, −*z*+2.
